# Microbiome–metabolome interaction reveals rhizosphere ecological, bioactive, and antioxidant responses of *Paeonia lactiflora* Pall. to intercropping and harvest timing

**DOI:** 10.3389/fmicb.2026.1934798

**Published:** 2026-09-03

**Authors:** Shuaiwen Yin, Cheng Jing, Caiying Dai, Xinjie Zhou, Jiahao Ai, Huimin Sun

**Affiliations:** 1 School of Life Sciences, Jinggangshan University, Ji’an, China; 2 Key Laboratory of Jiangxi Province for Biological Invasion and Biosecurity, School of Life Sciences, Jinggangshan University, Ji’an, China; 3 Key Laboratory of Jiangxi Province for Functional Biology and Pollution Control in Red Soil Regions, School of Life Sciences, Jinggangshan University, Ji’an, China

**Keywords:** active ingredients, antioxidant activity, metabolome, microbiome, *Paeonia lactiflora* Pall. (*P. lactiflora*)

## Abstract

*Paeonia lactiflora* Pall. (*P. lactiflora*) is a medicinal plant with considerable economic value. During its growth, root secondary metabolism and rhizosphere microbes interact closely; however, no systematic study has examined microbiome and metabolome dynamics under different planting systems and harvest times. To address this, we employed amplicon sequencing for microbial diversity and untargeted metabolomics for metabolite profiling. The datasets were then integrated, and correlation analysis was performed. We measured paeoniflorin by high-performance liquid chromatography (HPLC), total phenolic content by the Folin–Ciocalteu method, total flavonoid content by the NaNO_2−_Al(NO3)_3_ method, and antioxidant activity by DPPH, ABTS, hydroxyl radical scavenging, and FRAP assays. The results revealed that *Proteobacteria* was the most abundant phylum in the bacterial community, and among classified genera, *Subgroup_2* exhibited the highest relative abundance. In the fungal community, *Ascomycota* dominated at the phylum level, while *Coniosporium* was the most abundant genus. Metabolomic analysis revealed clear separation among sample groups by principal component analysis. Four pairwise comparisons identified differential metabolites predominantly classified as lipids, phenylpropanoids, and polyketides. Functional enrichment analysis indicated that these differences were mainly associated with metabolic pathways, particularly amino acid metabolism, secondary metabolite biosynthesis, and ABC transporters. Furthermore, monoculture with late-stage harvest (CS-4) represented the optimal strategy for maximizing medicinal quality. Under intercropping, mid-stage harvest (TZ-2/TZ-3), with adjustments based on annual climatic conditions, is recommended to balance quality and ecological benefits. Collectively, these findings reveal differences in *P. lactiflora* across planting systems and harvest periods, providing a theoretical basis for the optimization of cultivation strategies.

## 1 Introduction

The pharmacological activities of medicinal plants stem from bioactive compounds accumulated in their tissues, which are primarily secondary metabolites synthesized through plant secondary metabolic pathways. *Paeonia lactiflora* (*P. lactiflora*), a species of the genus Paeonia, has historically been used as a traditional Chinese medicine and has a wide range of clinical applications. According to traditional Chinese medicine theory, it can be used to treat blood deficiency and jaundice, nourish the blood and regulate menstruation, astringe yin to stop sweating, soften the liver to relieve pain, alleviate limb contracture and headaches, and calm liver yang (Lv et al., 2024). The plant also has anti-inflammatory, antispasmodic, analgesic, antibacterial, and laxative effects, and can help with acute liver injury and immune regulation (Yu et al., 2025). Its main active ingredients are paeoniflorin, total phenols, and total flavonoids. The production and buildup of these metabolites are affected by several factors, including the planting method, harvest time, and soil microbial environment.

The planting method has a strong influence on the growth, development, and quality of medicinal plants. Intercropping is an ecologically strong farming practice that can change the soil microenvironment, aid nutrient cycling, and alter the rhizosphere microbial communities, thus affecting the plant’s physiological and metabolic processes. Previous studies have confirmed that intercropping medicinal plants with fruit trees (e.g., walnut–tea intercropping) improves soil nutrient content, enzyme activity, and microbial diversity, thereby facilitating plant growth (Bai Y.-C. et al., 2022). Intercropping systems have also been reported to enhance the soil ecological environment and photosynthetic capacity, resulting in higher yields (Yang et al., 2021). Compared with monoculture, intercropping provides more efficient land use and contributes to improvements in soil quality and biodiversity (Lokossou et al., 2026; Singh et al., 2018). Tea plants are widely cultivated as a temperate crop in southern China and are characterized by moderate tree size and light requirements (Hao et al., 2022). Under *P. lactiflora*–tea plant intercropping, the tea canopy provides shade, which helps to regulate the temperature and humidity in the field. In addition, tea plant roots can interact with the rhizosphere microbes of *P. lactiflora*, potentially improving the quality of the latter. However, research on *P. lactiflora*–tea plant intercropping is lacking, and the impacts of this system on the rhizosphere microbial community and metabolite buildup in *P. lactiflora* remain unclear.

Metabolomics helps us understand metabolite biosynthesis in medicinal plants by comparing the metabolite profiles, analyzing metabolic pathways, and building metabolic networks (Fraga-Corral et al., 2022; Hao et al., 2025). Rhizosphere microorganisms are closely linked to plant growth (Ganugi et al., 2023). They can promote plant development through symbiotic relationships with the roots (Wu et al., 2025), and also improve disease resistance, nutrient use efficiency, and tolerance to both abiotic and biotic stresses (Trivedi et al., 2020; Bai B. et al., 2022; Bai Y.-C. et al., 2022; Zhang J. et al., 2019; Zhang S. et al., 2019; Chen et al., 2024). Therefore, rhizosphere microorganisms play an essential role in helping plant growth and development (Li et al., 2021).

Antioxidant activity refers to the ability of a substance to scavenge free radicals or inhibit oxidative processes, thereby delaying or preventing oxidative reactions. Compounds with strong antioxidant activity can protect against oxidative damage, which aids in maintaining cells intact and balanced (Nwozo et al., 2023). Antioxidant activity varies among *P. lactiflora* varieties, and similar variation has also been reported in other medicinal species, including hawthorn (Konyalioglu et al., 2017).

Harvest time is also a key influencing factor of medicinal plant quality. The production and accumulation of secondary metabolites vary across growth stages, and thus harvesting at the correct time is critical to ensure plant quality. Paeoniflorin, a water-soluble monoterpene glycoside, is an important marker for evaluating the quality and therapeutic effect of *P. lactiflora* (Shi et al., 2015). However, it remains unclear whether intercropping alters the period of high bioactive compound accumulation or simply enhances compound levels at specific harvest stages. To date, no systematic comparison has been conducted to evaluate the effects of different planting systems across multiple harvest periods.

In this study, we used 16S rRNA and ITS amplicon sequencing combined with untargeted metabolomics to investigate changes in the microbial and metabolic profiles of *P. lactiflora* under different planting systems and harvest periods. We also performed integrative analyses of microbe–metabolite interactions, quantified bioactive compounds (paeoniflorin, total phenols, and total flavonoids), and tested antioxidant capacity through DPPH, ABTS, FRAP, and hydroxyl radical (·OH) scavenging assays. This provides a theoretical foundation and practical guidance for the high-quality and efficient cultivation of *P. lactiflora*.

## 2 Materials and methods

### 2.1 Sample preparation

*Paeonia lactiflora* samples were collected from Jinggangshan City, Jiangxi Province, China, in September 2025. We sampled plants under two systems—*P. lactiflora*–tea plant intercropping and *P. lactiflora* monoculture—and four harvest times. Three biological replicates were set up, respectively, for the intercropping (TZ) and monoculture (CS) groups. Sampling began in September 2025 and was conducted at 20-day intervals. This resulted in the four harvest time points TZ-1/CS-1, TZ-2/CS-2, TZ-3/CS-3, and TZ-4/CS-4, corresponding to active root growth, early root tuber thickening, rapid root tuber thickening, and root maturation to early senescence, respectively. The intercropping and monoculture plots were established in adjacent fields, with each plot independently partitioned and spatially separated within the experimental area. All agronomic practices—including soil type, irrigation, fertilization, and pest management—were strictly uniform across all plots. This design minimized confounding effects from other factors, ensuring that observed differences could be primarily attributed to the planting system and harvest time.

At each sampling time, we randomly selected three healthy and similar-sized *P. lactiflora* plants from the same batch. The root components and rhizosphere soil of these plants were collected, and the latter was stored in a −80 °C freezer for microbial analysis (Lin et al., 2023). For root metabolite measurements and the bioactivity tests, the root samples were washed with distilled water, dried, and then cut into 1-cm pieces. The pieces were then ground into powder using liquid nitrogen. The resulting powder was placed into cryovials and stored at −80 °C until analysis of paeoniflorin, total phenols, total flavonoids, and antioxidant activity (Deng et al., 2024).

### 2.2 Soil microbial extraction and sequencing

Each mixed soil sample (200–500 mg) was transferred into a sterile 2 mL tube, followed by the addition of 1 × PBS solution. The samples were then vortexed until thoroughly mixed. Following this, the mixture was centrifuged at 10,000 rpm for 3 min at room temperature, and the supernatant was discarded. To remove any residual liquid, the open tubes were inverted onto absorbent paper for 1 min and then placed in a metal bath at 55 °C for 10 min to ensure complete evaporation before subsequent procedures. Total DNA was extracted from these processed samples using a commercial DNA extraction kit. ITS and 16S amplicon libraries were constructed, followed by high-throughput sequencing on an Illumina MiSeq platform. Extracted total DNA was employed as a PCR template, and target-specific primers were used for amplification. The resulting amplicons were pooled, eluted with Tris–HCl, and separated on a 2% agarose gel to assess fragment size and quality. The amplicons were then quantified accurately and subjected to subsequent processing steps (Li et al., 2020).

### 2.3 Metabolome extraction and sequencing

#### 2.3.1 Metabolite preparation

A total of 40 mg of ground plant material was transferred into a 2-mL centrifuge tube. Following this, 300 μL of a cooled mixture (methanol:acetonitrile:water = 2:2:1) containing 5 ppm of 2-chlorophenylalanine was added, along with two steel beads. The mixture was vortexed for 30 s and homogenized using a tissue grinder at 55 Hz for 60 s. This grinding step was repeated once. The homogenate was then sonicated for 10 min, frozen at −20 °C for 30 min, and centrifuged at 12,000 rpm for 10 min at 4 °C. The resultant supernatant was filtered through a 0.22-μm membrane, and the filtrate was collected into a sample vial for liquid chromatography–mass spectrometry (LC–MS) analysis, following the method of Want et al. (2013).

#### 2.3.2 Liquid chromatographic conditions

The samples were analyzed in a randomized order. Quality control (QC) and blank samples were injected after every 8–10 runs. The QC sample was prepared by mixing equal volumes of all test solutions and used to maintain the relative standard deviation below 30%. The separation was performed using an ACQUITY UPLC HSS T3 column (2.1 × 100 mm, 1.8 μm) at 40 °C with a flow rate of 0.4 mL/min. The mobile phases consisted of 0.1% formic acid in water (A) and 0.1% formic acid in acetonitrile (B). The gradient program was as follows: 0–1 min, 2% B; 1–9 min, 2–98% B; 9–12 min, 98% B; 12.1 min, returned to 2% B; and 12.1–15 min, column re-equilibration at 2% B (Alseekh et al., 2021).

#### 2.3.3 Mass spectrometry conditions

Mass spectrometry analysis was performed on an Orbitrap Exploris 120 mass spectrometer equipped with an electrospray ionization source operating in both positive and negative ion modes— and spray voltages set to +3.5 kV and −3.0 kV, respectively. The capillary was maintained at 320 °C, and the sheath and auxiliary gas flow rates were set to 40 and 10 arbitrary units, respectively. Full-scan MS1 spectra were acquired over the *m/z* range of 70–1,000 at a 60,000 resolution. Data-dependent acquisition was subsequently performed for MS2 analysis, with the 10 most intense precursor ions selected for fragmentation at a 15,000 resolution. Higher-energy collisional dissociation was performed using a normalized collision energy of 30% and a dynamic exclusion time of 4 s. Raw data were processed using MS-DIAL (version 4.9.221218). Using this software, peak extraction, alignment, filtering, metabolite identification, and other preprocessing steps were performed. Peak intensities were normalized using either total ion current or an internal standard, followed by Pareto scaling. Missing values were imputed using the k-nearest neighbor method or semi-minimum estimation. Differential metabolites were screened using the criteria |log₂(fold change)| > 1 and a Benjamini–Hochberg corrected q-value < 0.05. Tentative annotation was performed according to Schmid et al. (2023) by matching accurate MS1 masses and MS2 spectra against our in-house database, as well as the NIST and GNPS libraries. The annotation confidence was evaluated using spectral matching scores and retention index values.

### 2.4 Determination of active ingredients

For paeoniflorin determination, 1.00 g of sample was transferred into a 50-mL centrifuge tube and extracted with 25 mL of 60% methanol by ultrasonification for 90 min. The extract was centrifuged at 4000 rpm for 5 min, and the supernatant was collected and diluted to 25 mL in a volumetric flask. The solution was mixed thoroughly, filtered through a 0.22-μm membrane, and the filtrate was collected for analysis. Paeoniflorin was quantified using a Thermo Fisher U3000 high-performance liquid chromatography (HPLC) system with an LP-C18 column (4.6 mm × 250 mm, 5 μm). The mobile phase consisted of 0.1% phosphoric acid in water and acetonitrile. The column temperature was maintained at 30 ± 2 °C, the detection wavelength was set at 230 nm, the flow rate was 1.0 mL/min, and the injection volume was 2 μL (Ma et al., 2018).

Total phenolic content (TPC) was determined using a modified Folin–Ciocalteu method according to He et al. (2016). A standard solution was prepared by dissolving 0.025 g of gallic acid in distilled water and diluting the solution to 100 mL in a volumetric flask to prepare the standard solution. Aliquots of the solution (0, 1, 2, 3, 4, 5, 6, and 7 mL) were transferred into separate 25-mL stoppered tubes and diluted to volume with distilled water. An aliquot (1 mL) of the diluted sample was mixed with 1 mL of Folin–Ciocalteu reagent, 3 mL of 12.5% sodium carbonate solution, and 5 mL of distilled water. The tubes were maintained in a 30 °C water bath for 1 h, and the absorbance was measured. A standard calibration curve was created by plotting the gallic acid concentration (x) against absorbance (y), and the linear regression equation was used to calculate TPC according to Equation 1:


$$W=\frac{\left(C\times V\times N\right)}{M}\times 100\%$$ (1)

where *C* denotes the measured concentration (mg/mL); *V* is the extraction solvent volume (mL); *N* represents the dilution factor; and M is the raw material mass (g).

Total flavonoid content (TFC) was determined using a modified sodium nitrite–aluminum nitrate method described in Li et al. (2012). A rutin stock solution (0.1 mg/mL) was prepared by dissolving 10 mg of rutin in 95% ethanol and diluting the solution to 100 mL in a volumetric flask. Standard solutions were prepared by mixing 0–5 mL of the rutin solution with 95% ethanol to a total volume of 5 mL. To each standard solution, 0.3 mL of 5% sodium nitrite was added, and the mixture was allowed to stand for 5 min. Subsequently, 0.3 mL of 10% aluminum nitrate was added, and the solution was allowed to stand for another 6 min. Following this, 4 mL of 4% sodium hydroxide was added, and the reaction mixture was diluted to 10 mL with distilled water. The absorbance was measured at 510 nm. A standard calibration curve was constructed by plotting rutin concentration (Y, mg/mL) against the absorbance (A). For sample analysis, 5 mL of the test solution was transferred into a 25-mL graduated tube and treated using the same procedure as described above. The flavonoid extraction yield (*W*) was calculated using Equation 1.

### 2.5 Determination of antioxidant activity

#### 2.5.1 Determination of DPPH free radical scavenging ability

The DPPH radical scavenging activity was determined using a modified method according to Eruygur et al. (2019). Three reaction mixtures were prepared: a blank consisting of 100 μL of 0.2 mmol/L DPPH solution and 100 μL of anhydrous ethanol; a sample mixture consisting of 100 μL of DPPH solution and 100 μL of the sample solution; and a sample control consisting of 100 μL of ethanol and 100 μL of sample solution. All reaction mixtures were incubated in the dark at room temperature for 30 min. Trolox was used as the positive control. Absorbance was measured at 517 nm and recorded as *A₀* (blank), *A₁* (sample), and *A₂* (control). The DPPH radical scavenging rate (*S*) was calculated using Equation 2.


$$S=[1-\frac{\left(A1-A2\right)}{A0}]\times 100\%$$ (2)

#### 2.5.2 Determination of ABTS free radical scavenging ability

The ABTS radical scavenging activity was determined using a modified method according to Dong et al. (2014). A 7 mmol/L ABTS stock solution was prepared in 2.45 mmol/L potassium persulfate and allowed to stand in the dark for 16 h. The stock solution was diluted with deionized water until it reached an absorbance of 0.70 ± 0.01 at 734 nm. Three reaction systems were prepared: a blank control consisting of 100 μL of ABTS working solution and 100 μL of distilled water; a sample mixture consisting of 100 μL of ABTS and 100 μL of sample solution; and a sample control consisting of 100 μL of distilled water and 100 μL of sample solution. All mixtures were incubated in the dark for 30 min. Trolox was used as the positive control. The absorbance was measured at 734 nm and recorded as *A₀* (blank), *A₁* (sample), and *A₂* (control). The ABTS radical scavenging rate was calculated using Equation 2.

#### 2.5.3 Determination of hydroxyl radical scavenging ability

Hydroxyl radical (·OH) scavenging activity was determined using a modified method according to Ding et al. (2021). Three reaction systems were prepared: a blank control, a sample mixture, and a sample control. The blank control consisted of 1 mL of 6 mmol/L FeSO₄, 1 mL of 6 mmol/L salicylic acid, and 1 mL of distilled water. After incubation in the dark for 10 min, 1 mL of H₂O₂ was added. The sample mixture consisted of 1 mL of FeSO₄, 1 mL of salicylic acid, and 1 mL of sample solution. After incubation in the dark for 10 min, 1 mL of 6 mmol/L H₂O₂ was added, and the mixture was incubated in a 37 °C water bath for 30 min. The sample control was prepared in the same manner as the sample mixture, except that 1 mL of distilled water was used instead of the H₂O₂ solution. Trolox was used as the positive control. Absorbance was measured at 510 nm and recorded as *A₀* (blank), *A₁* (sample), and *A₂* (correction). The ·OH scavenging rate was calculated using Equation 2.

#### 2.5.4 Determination of ferric reducing antioxidant power reduction ability

Ferric reducing antioxidant power (FRAP) was determined using modified methods according to Saimaiti et al. (2022) and Song et al. (2010). The FRAP working solution was prepared by mixing 300 mmol/L sodium acetate buffer, 10 mmol/L 2,4,6-tris(2-pyridyl)-s-triazine (TPTZ) solution, and 20 mmol/L FeCl₃ solution at a 10: 1: 1 volume ratio, followed by incubation at 37 °C for 10 min. A Trolox stock solution (1.0 mg/mL) was prepared in 95% ethanol and diluted to obtain standard solutions of 0.05, 0.10, 0.15, 0.20, and 0.25 mg/mL. For each standard, 0.1 mL of Trolox solution was mixed with 3 mL of FRAP working solution and incubated at 37 °C for 10 min, after which the absorbance was measured at 593 nm. A standard calibration curve was constructed by plotting Trolox concentration against absorbance.

For sample analysis, 100 μL of sample solution was mixed with 3 mL of FRAP working solution and incubated at 37 °C for 10 min. Trolox was used as the positive control. The absorbance was measured at 593 nm and recorded as *A₁*. A blank control was prepared by replacing the sample with the corresponding solvent, and the absorbance was recorded as *A₀.* The ferric reducing ability (*T*) was determined using Equation 3.


T=A1−A0 (3)

### 2.6 Statistical analysis

Statistical analyses were performed using SPSS 21.0 (IBM SPSS Inc., United States). Alpha diversity indices (Chao1 and Shannon) were calculated using the R (v4.6.1). to assess microbial richness and diversity. Principal component analysis (PCA) was employed to evaluate microbial community structure and visualize the metabolomic data distribution. Differences in community composition were assessed via permutational multivariate analysis of variance based on Bray–Curtis dissimilarity. Linear discriminant analysis Effect size (LEfSe) was used to identify differentially abundant microbial taxa between groups. Differential metabolites were analyzed using the t-test. For multi-omics integration, differential metabolites were mapped to Kyoto Encyclopedia of Genes and Genomes (KEGG) pathways to identify relevant metabolic pathways. Correlation network analysis was performed in R (v4.6.1) and visualized with Gephi (v0.9.5). All antioxidant activity data were expressed as the mean ± standard deviation (SD; *n* = 3). Two-way ANOVA was conducted to evaluate the effects of the planting system, harvest time, and their interaction. Bar charts were generated using Origin 2024.

## 3 Results

### 3.1 Effects of different planting systems and harvest time on soil microorganisms

#### 3.1.1 Taxonomic composition analysis

Illumina sequencing was performed to characterize the rhizosphere bacterial and fungal communities. Bacterial sequencing yielded 2,681,075 raw reads, which, after splicing, quality control, and filtering, generated 1,560,050 valid reads (Supplementary Table 1). Fungal sequencing produced 2,708,609 raw reads, resulting in 2,003,623 valid reads after quality filtering (Supplementary Table 2).

**Figure 1:**
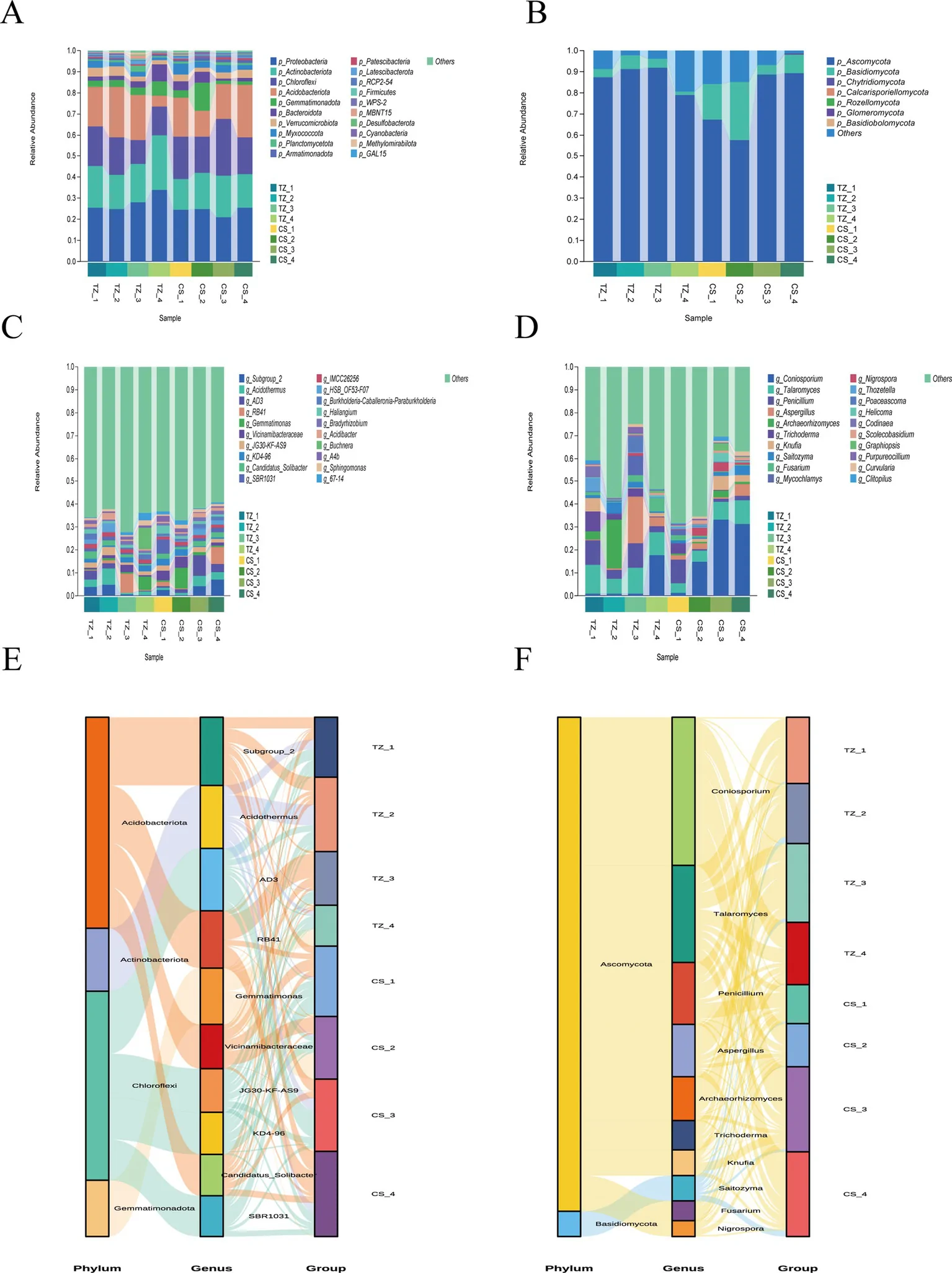
Relative abundance of rhizosphere microbial communities. **(A)** Bacterial phylum-level composition. **(B)** Fungal phylum-level composition. **(C)** Bacterial genus-level composition. **(D)** Fungal genus-level composition. **(E)** Integrated analysis of bacterial abundance from phylum to genus level. **(F)** Integrated analysis of fungal abundance from phylum to genus level.

We analyzed the composition of rhizosphere bacterial and fungal communities at both the phylum and genus levels. At the bacterial phylum level (Figure 1A), *Proteobacteria*, *Actinobacteriota*, *Chloroflexi*, *Acidobacteriota*, *Gemmatimonadota*, *Bacteroidota*, *Verrucomicrobiota*, and *Myxococcota* collectively accounted for approximately 95% of the total bacterial relative abundance, with *Proteobacteria* being the most dominant phylum (Supplementary Table 3). In the intercropping (TZ) group, the abundances of *Proteobacteria*, *Actinobacteriota*, and *Acidobacteriota* remained consistently high, while *Chloroflexi* gradually declined with harvest progression, indicating a relatively stable community structure. In contrast, the monoculture (CS) group exhibited a marked increase in *Chloroflexi* abundance, accompanied by corresponding decreases in *Proteobacteria* and *Acidobacteriota*, with pronounced fluctuations in dominant taxa across harvest periods.

At the fungal phylum level (Figure 1B), *Ascomycota* was the absolute dominant phylum across all samples. Its relative abundance remained stable in the TZ group (80–92%), whereas in the CS group, it declined to 58% at CS-2 and fluctuated over time. In the TZ group, the coefficient of variation for *Ascomycota* abundance across harvest periods was only 0.33%, lower than the 22.59% observed in the CS group (Supplementary Table 4). This indicates greater temporal stability of the dominant fungal community under intercropping. *Basidiomycota*, the second most abundant phylum, was enriched in the CS group (peaking at CS-2), while its abundance was steadily suppressed at 5–10% in the TZ group. Both *Ascomycota* and *Basidiomycota* are important endophytic fungal taxa that form symbiotic associations with plants, promoting growth and health, enhancing stress resistance, and performing key ecological functions (Zhu et al., 2023).

At the bacterial genus level (Figure 1C), excluding unclassified taxa, *Subgroup_2*, *Acidothermus*, and *AD3* exhibited the highest relative abundances (Supplementary Table 5). Unclassified bacteria dominated all samples, accounting for 61.8–72.3% of the total. *Subgroup_2* consistently maintained a higher proportion in the TZ group compared to the CS group. At the fungal genus level (Figure 1D), except for unclassified fungi, *Coniosporium*, *Talaromyces*, and *Penicillium* were the most abundant classified genera (Supplementary Table 6). The TZ group was enriched in known functional genera such as *Trichoderma*, *Penicillium*, and *Talaromyces*, with the proportion of “Others” remaining stable at 24.99–57.30%, reflecting a relatively stable community structure. In contrast, the CS group showed a marked increase in *Coniosporium* abundance, with “Others” rising to 30.42–68.17%. This suggests that intercropping contributes to maintaining fungal community homeostasis.

The high proportion of unclassified taxa across all samples is likely attributable to the combined effects of database limitations and sequencing region constraints. Current microbial reference databases primarily contain sequences from known culturable or sequenced microorganisms, while the vast majority of microbial gene sequences in nature remain uncatalogued.

*Acidobacteriota* was determined as the core phylum at the bacterial level (Figure 1E). The TZ group was enriched in the dominant genera *Subgroup_2* and *Candidatus_Solibacter*, resulting in a more concentrated and stable community structure. In the CS group, *Acidobacteriota* showed a shift in community composition toward *AD3*. At the fungal level (Figure 1F), *Ascomycota* was identified as the dominant phylum. The TZ group was enriched in the functional genera *Talaromyces* and *Trichoderma*, demonstrating a more concentrated and stable structure, while the CS group displayed enrichment of *Coniosporium*, with a more dispersed community structure.

#### 3.1.2 α-diversity and β-diversity

**Figure 2:**
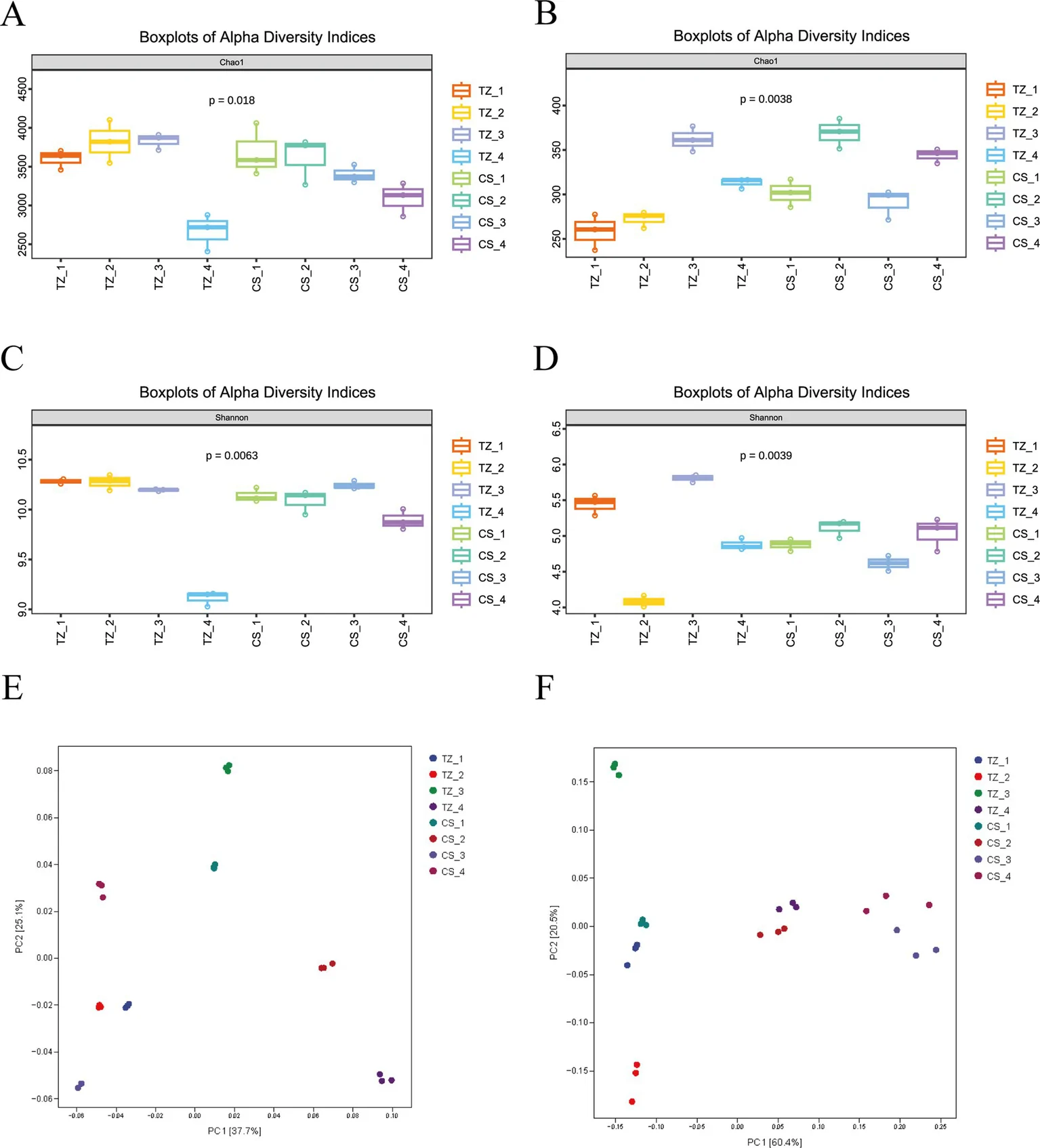
Alpha diversity and beta diversity. **(A,B)** Bacterial and fungal Chao1 index. **(C,D)** Bacterial and fungal Shannon index. **(E)** PCA plot of bacterial communities. **(F)** PCA plot of fungal communities.

Figures 2A–D depicts the Shannon and Chao1 indices, which reflect microbial diversity and richness, respectively. The figure reveals significant differences between the two planting systems. Alpha diversity analysis showed markedly higher Chao1 and Shannon index values in the intercropping (TZ) group compared to the monoculture (CS) group. This suggested that intercropping boosted the richness and diversity of the soil bacteria. However, the opposite was observed for fungi, namely higher Chao1 and Shannon indices were observed in the CS group. This suggests that the single-crop system was more effective in increasing fungal diversity.

Within-group community dissimilarity calculated using Bray–Curtis distances revealed that the TZ group exhibited clustered intra-group distances with a narrow dispersion range (Supplementary Figure 1). In contrast, the CS group displayed a wide span of intra-group distances with pronounced temporal fluctuations. In terms of overall community structure, this demonstrates that intercropping effectively reduces temporal fluctuations and maintains community homeostasis.

Based on amplicon sequence variant classification, distinct differences in species composition were observed between the TZ and CS groups (Supplementary Figure 2). PCA further confirmed this separation (Figures 2E,F). In particular, for the bacterial community, PC1 and PC2 accounted for 37.7 and 25.1% of the total variation, respectively, while for the fungal community, PC1 and PC2 explained 60.4 and 20.9% of the variation, respectively. These results indicate differences in the bacterial and fungal community structures between the TZ and CS groups.

#### 3.1.3 Significant difference analysis of microbial flora in rhizosphere soil

**Figure 3:**
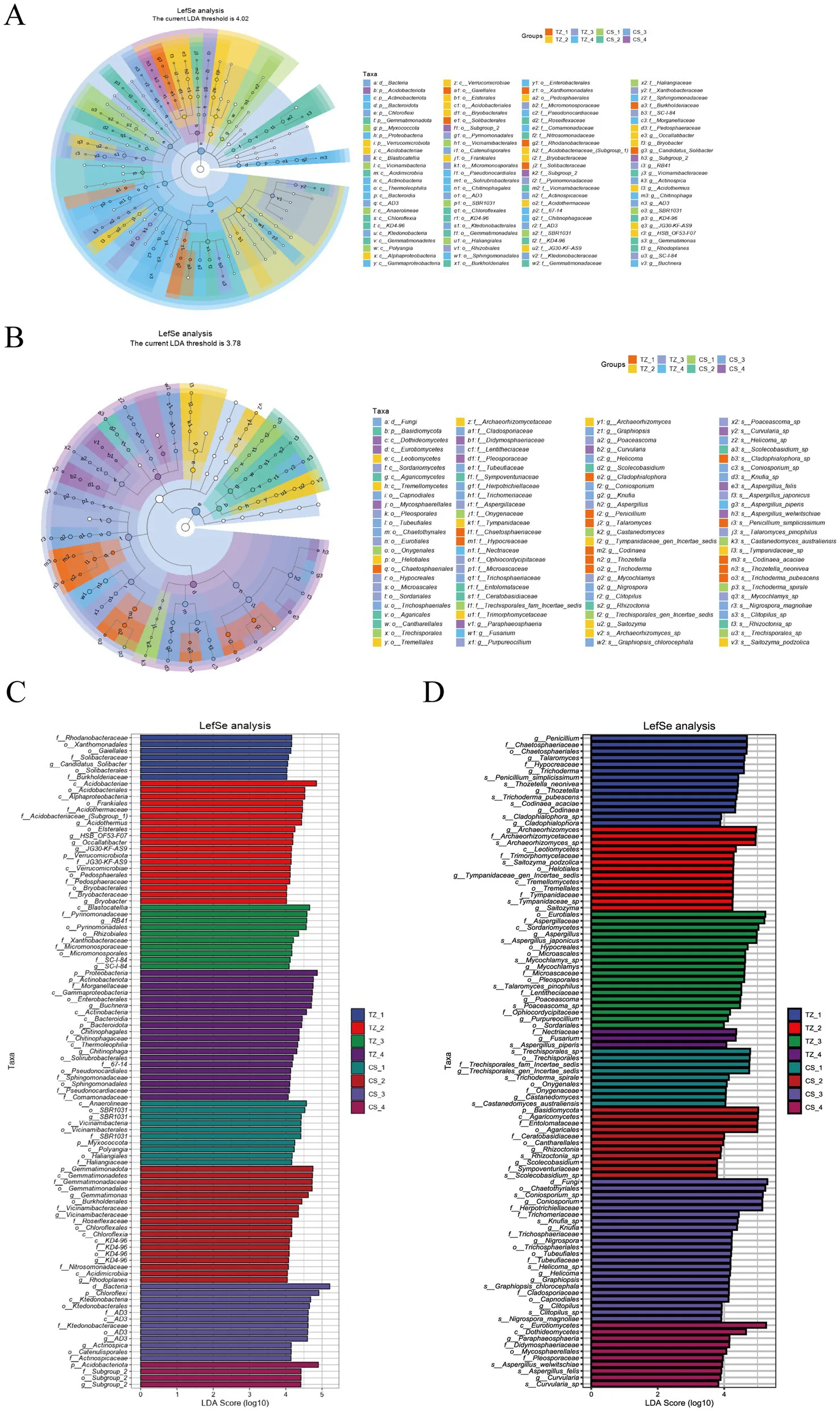
LEfSe differential analysis of soil bacterial and fungal communities. **(A)** Cladogram of the bacterial community. **(B)** Cladogram of the fungal community. **(C)** LDA score histogram of the bacterial community. **(D)** LDA score histogram of the fungal community.

LEfSe analysis revealed differential enrichment of bacterial and fungal taxa between groups, with linear discriminant analysis score thresholds of 4.02 and 3.78 for bacteria and fungi, respectively. At the bacterial level (Figures 3A,C and Supplementary Table 7), the TZ group was characterized by *Acidobacteriota* as the core phylum, enriched in the genera *Subgroup_2* and *Acidothermus*, *Candidatus_Solibacter*, and exhibited a stable community structure across harvest periods. In contrast, the CS group was enriched in *Chloroflexi* with oligotrophic genera (*AD3* and *RB41*), showing weaker stability. At the fungal level (Figures 3B,D and Supplementary Table 8), the TZ group was enriched in genera *Talaromyces*, *Penicillium*, *Trichoderma*, and *Archaeorhizomyces*, with a stable structure. The CS group displayed a marked increase in *Basidiomycota* at CS-2 and dominance of *Coniosporium* at CS-3, indicating pronounced community fluctuations.

*Trichoderma* is a well-known biocontrol agent against pathogens such as *Fusarium oxysporum*, *Rhizoctonia solani*, and *Botrytis cinerea* (Redda et al., 2018). *Talaromyces*, belonging to *Ascomycota*, promotes plant growth and health through symbiotic associations (Zhu et al., 2023).

The findings indicate that intercropping buffered harvest-induced disturbances and maintained microbial community stability by stabilizing core functional groups across taxonomic levels, whereas the monoculture community was more sensitive to temporal variation.

#### 3.1.4 Potential function prediction of rhizosphere soil microorganisms

**Figure 4:**
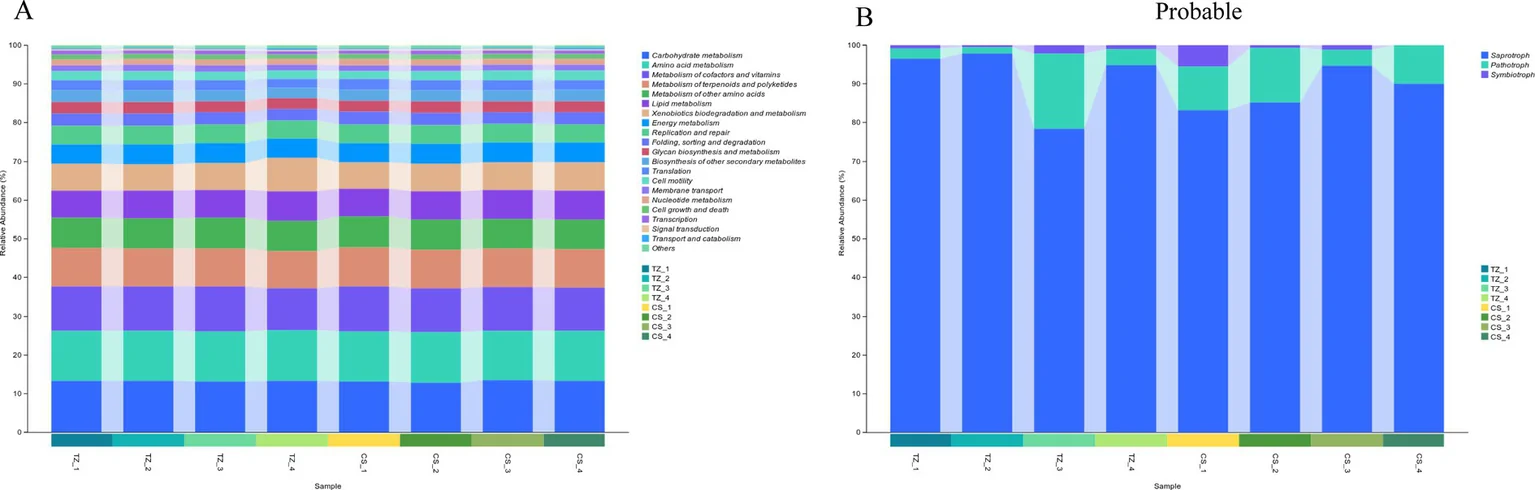
Functional prediction analysis of soil bacterial and fungal communities. **(A)** KEGG metabolic function of soil bacteria. **(B)** FUNGuild ecological function analysis of fungi.

Based on PICRUSt2 functional prediction, the KEGG metabolic pathways of soil bacteria in the TZ and CS groups were analyzed. KEGG annotation identified numerous level-2 pathways, and detailed pathway information is provided in Supplementary Table 9. A bar chart (Figure 4A) was constructed to depict the functional composition of the annotated pathways. The results showed that the pathways for terpenoid and polyketide metabolism, amino acid metabolism, carbohydrate metabolism, lipid metabolism, and the metabolism of cofactors and vitamins were among the most abundant functional categories. The functional structures of all samples were in close agreement, indicating high functional redundancy and strong stability of the soil bacterial community.

FUNGuild assignment revealed three core functional guilds: Saprotroph, Pathotroph, and Symbiotroph (Supplementary Table 10). Saprotrophs dominated all samples (85–99.5%, Figure 4B), playing key roles in organic matter decomposition and nutrient cycling. Pathotrophs were less abundant in intercropping (0.5–2.0%) than in monoculture (14.5–15.0%), suggesting intercropping may suppress pathotroph colonization. In contrast, Symbiotrophs showed no marked differences between groups. Predictions at other confidence levels are provided in Supplementary Figure 3. As FUNGuild predictions reflect only potential functions, the inferred reduction in disease risk under intercropping requires experimental validation.

### 3.2 Effects of planting systems and harvest time on the metabolome

**Figure 5:**
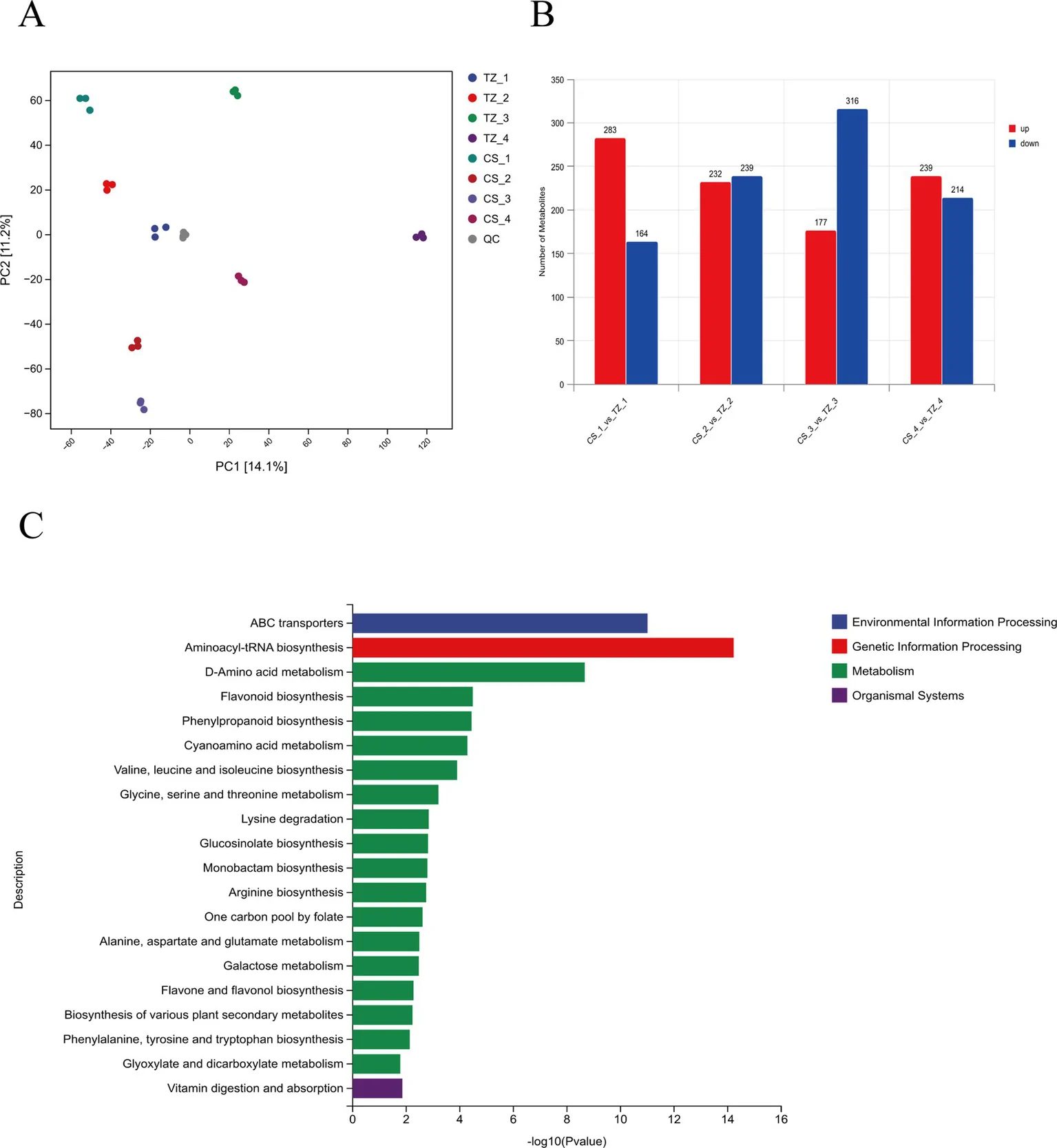
Changes in root metabolites. **(A)** PCA plot. **(B)** Statistics of differential metabolites. **(C)** KEGG pathway enrichment analysis.

The PCA performed on the 24 metabolites separated the samples into eight groups. PC1 and PC2 accounted for 14.1 and 11.2% of the total variance, respectively (Figure 5A). Pairwise comparisons between the TZ and CS groups at the four harvest time points (CS-1 vs. TZ-1 through CS-4 vs. TZ-4) yielded 447, 471, 493, and 453 differential metabolites, including 283/164, 232/239, 177/316, and 239/214 upregulated/downregulated metabolites, respectively (Figure 5B). Volcano plots were generated for each of the four treatment pairs to visualize the fold-change magnitudes and statistical significance of differential metabolites across comparison groups (Supplementary Figure 4). In addition, the top 30 core differential metabolites from each comparison were selected and compiled, including fold changes and putative functions (Supplementary Table 11). Beyond compositional differences, Venn analysis identified both shared and treatment-specific metabolic signatures, with 88, 82, 92, and 88 unique metabolites detected exclusively in each treatment contrast, indicating that cultivation regime and harvesting phenophase collectively influence root metabolic profiles (Supplementary Figure 5). The differential metabolites were classified into several major categories, including lipids, phenylpropanoids and polyketides, benzenoids, organoheterocyclic compounds, and lignans/neolignans (Supplementary Figure 6).

KEGG enrichment annotated 20 pathways spanning four principal categories (Figure 5C). The metabolic category included pathways related to amino acid, secondary metabolite, carbohydrate, lipid, one-carbon, and organic acid metabolism. Genetic information processing was represented by aminoacyl-tRNA biosynthesis, environmental information processing by ABC transporters, and organismal systems by vitamin digestion/absorption. These findings indicate that the influence of different treatments on root metabolism is predominantly exerted on primary and secondary metabolic routes, with amino acid metabolism, secondary metabolite biosynthesis, and fundamental substance-transport pathways exhibiting the most obvious enrichment.

### 3.3 Combined analysis of microbiome and metabolome

To investigate endophyte–metabolite interactions and their effects on the metabolic profiles of *P. lactiflora* under different planting systems and harvest periods, we performed multi-omics correlation analysis using Pearson correlation (|R| > 0.6, *p* < 0.05), focusing on flavonoids, terpenoids, phenolics, organic acids, alkaloids, and their associations with core endophytic taxa.

**Figure 6:**
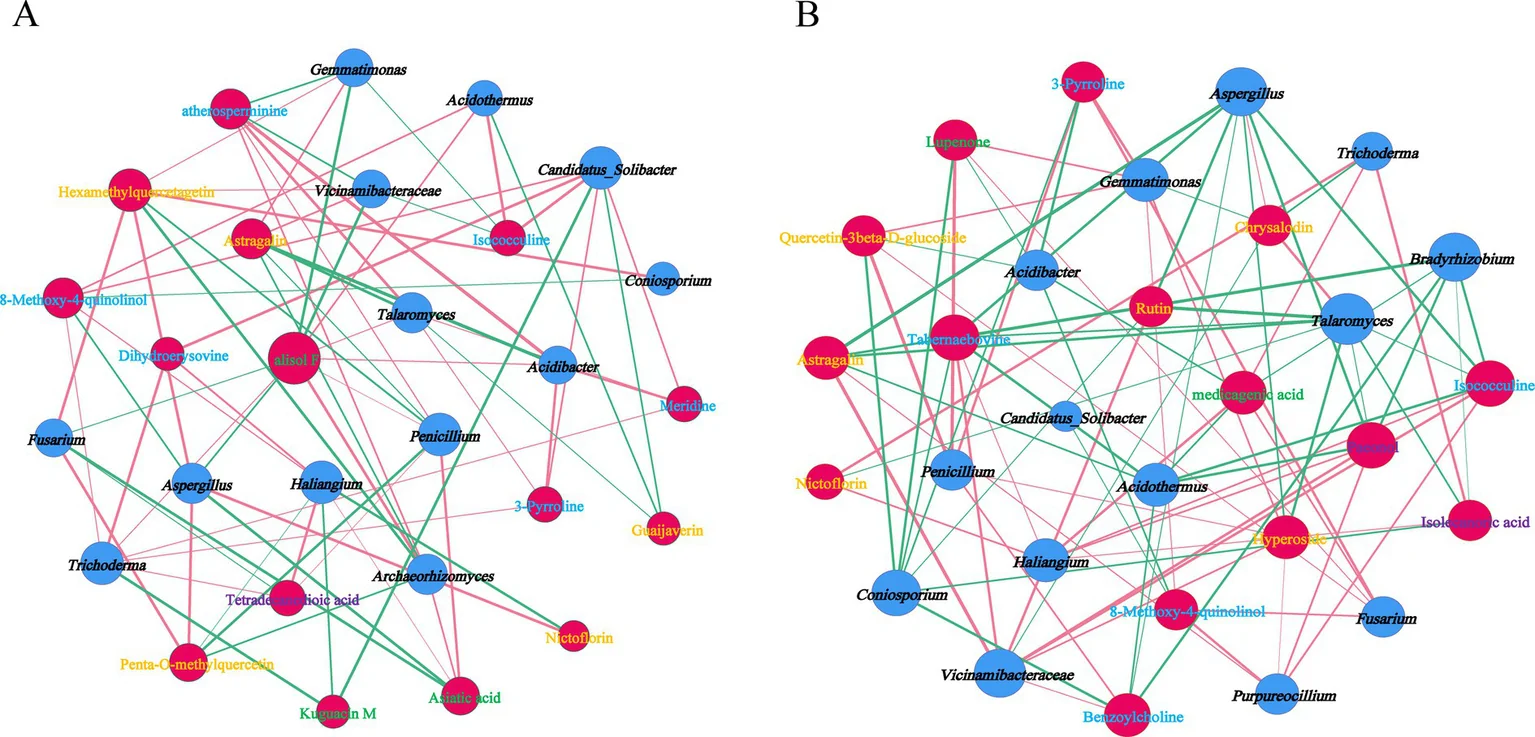
Co-occurrence network of differential microbial communities at genus level and metabolites. (Pearson; *R* > 0.6, *p* < 0.05) **(A)** TZ group **(B)** CS group. Microbes are represented by blue nodes, and metabolites are marked with red nodes (blue: alkaloids; green: terpenoids; yellow: flavonoids; purple: phenolics and organic acids). Red lines indicate positive correlations, green lines represent negative correlations, and the size of each circle reflects the degree of connectivity.

Under the two cropping regimes, plant secondary metabolites established intricate and significant interaction networks with both rhizobacteria and fungi. Notably, cropping systems directionally steered rhizosphere microbial co-occurrence patterns by altering root metabolite accumulation profiles. Correlation analysis identified 15 microbial genera significantly linked to metabolite variations, including *Acidothermus, Gemmatimonas, Vicinamibacteraceae, Candidatus_Solibacter, Haliangium, Bradyrhizobium, Acidibacter, Coniosporium, Talaromyces, Penicillium, Aspergillus, Archaeorhizomyces, Trichoderma, Fusarium, and Purpureocillium*. In the TZ group, *Acidothermus* exhibited significant correlations with four metabolites, including three positive correlations (8-Methoxy-4-quinolinol, alisol F, and Isococculine) and one negative correlation (Guaijaverin). *Candidatus_Solibacter* was correlated with seven metabolites, comprising five positive correlations (8-Methoxy-4-quinolinol, Dihydroerysovine, Isococculine, 3-Pyrroline, and Meridine) and two negative correlations (Kuguacin M and Guaijaverin) (Figure 6A). In the CS group, *Purpureocillium* was positively correlated with the five metabolites Isococculine, Paeonol, Hyperoside, 8-Methoxy-4-quinolinol, and Astragalin. *Vicinamibacteraceae* exhibited seven positive significant correlations (Hyperoside, Isococculine, Paeonol, Rutin, Tabernaebovine, Astragalin, and Benzoylcholine) and one negative significant correlation (Chrysalodin) (Figure 6B).

Among the metabolite classes, flavonoids presented the largest number of marked correlations with both rhizobacteria and fungi, representing core signaling hubs for rhizosphere microbial community structure (Li et al., 2025). For example, in the TZ group, highly enriched Hexamethylquercetagetin, Astragalin, Penta-O-methylquercitin, and Nictoflorin were positively correlated with *Fusarium, Aspergillus, Gemmatimonas, Vicinamibacteraceae, and Candidatus_Solibacter*. Conversely, in the CS group, enriched Quercetin-3β-D-glucoside, Astragalin, Nictoflorin, Chrysalodin, and Hyperoside exhibited strong positive correlations with *Penicillium, Haliangium, Vicinamibacteraceae, Fusarium, and Talaromyces*. The CS group exhibited a higher proportion of positive correlations within its interaction network.

In summary, secondary metabolite accumulation was significantly correlated with endophytic communities, suggesting potential interactions. However, this requires experimental validation.

### 3.4 Analysis of bioactive compounds and antioxidant activity

Bioactive components and antioxidant activity are core evaluation indicators for the quality of medicinal materials. Pairwise comparisons were performed between the TZ and CS groups at corresponding points (e.g., TZ-1 vs. CS-1, TZ-2 vs. CS-2) to avoid confounding the effects of planting system with those of harvest time.

**Figure 7:**
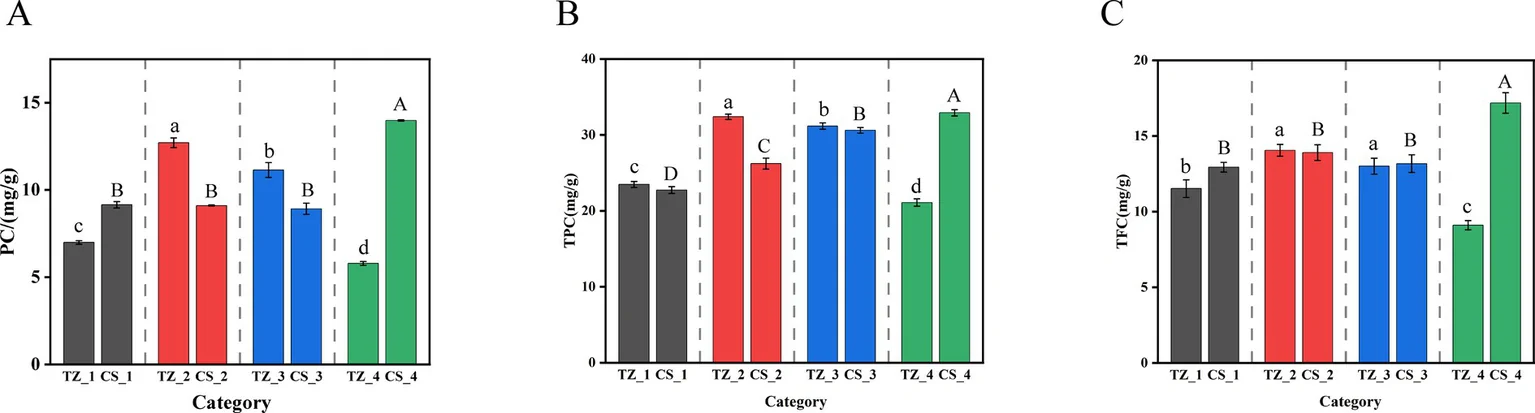
Contents of paeoniflorin, total phenolics, and total flavonoids. **(A)** Paeoniflorin content. **(B)** Total phenolic content. **(C)** Total flavonoid content. Data are presented as mean ± SD (*n* = 3); a–d: Different lowercase letters indicate significant differences among samples from different harvest periods within the TZ group (*p* < 0.05, one-way ANOVA); A, B: uppercase letters indicate significant differences among samples from different harvest periods within the CS group (*p* < 0.05, one-way ANOVA).

Paeoniflorin, the principal bioactive constituent of *P. lactiflora*, serves as a core quality indicator for the crude drug and its preparations (Wu et al., 2024). As shown in Figure 7A, paeoniflorin content varied considerably among the eight samples, with CS-4 exhibiting the highest value (13.99 mg/g) and TZ-4 the lowest (5.79 mg/g). The overall ranking was CS-4 > TZ-2 > TZ-3 > CS-1 > CS-2 > CS-3 > TZ-1 > TZ-4. In the TZ group, the paeoniflorin content initially increased and subsequently decreased, with the highest value observed at TZ-2—corresponding to the mid-harvest stage. Notably, the levels at TZ-2 and TZ-3 significantly exceeded those of the corresponding monoculture (CS) samples, while the converse was true for the early (TZ-1) and late (TZ-4) stages. In the CS group, paeoniflorin content increased throughout the study period, reaching a peak at the late harvest stage (CS-4), where it exceeded that observed in the TZ group. Based on these findings, the optimal harvest windows for maximizing paeoniflorin yield were the mid-harvest stage for the TZ group and the late-harvest stage for the CS group.

Total phenolic content contributed to the diverse pharmacological activities of *P. lactiflora*, and its accurate quantification facilitates deeper utilization of plant polyphenols (Withouck et al., 2023). As shown in Figure 7B, TPC values ranked from highest to lowest as follows: CS-4, TZ-2, TZ-3, CS-3, CS-2, TZ-1, CS-1, and TZ-4. In the TZ group, TPC initially increased and subsequently declined across the different harvest stages, reaching a peak at TZ-2. At this stage, the TPC value was higher than that observed in the CS group at the corresponding harvest stage. In contrast, the CS group demonstrated a sustained upward trend, with the highest TPC recorded at CS-4. Thus, the mid-harvest stage appears optimal for TPC accumulation in the TZ group, whereas late-harvest maximizes TPC levels in the CS group.

Flavonoids are a major class of active constituents in *P. lactiflora*, providing antibacterial, antiviral, and antioxidant effects (Shu et al., 2020). As presented in Figure 7C, TFC followed the order CS-4 > TZ-2 > CS-2 > CS-3 > TZ-3 > CS-1 > TZ-1 > TZ-4. Within the TZ group, TFC levels initially increased and subsequently declined, peaking at TZ-2. In addition, mid-stage TFC levels in the TZ group were similar to those in the CS group. A continuous upward trend was observed in the CS group, reaching a maximum at CS-4, exceeding the corresponding value in the TZ group. In summary, the mid-harvest stage is recommended for maximizing TFC in the TZ group, whereas the late-harvest stage is preferable for the CS group.

**Figure 8:**
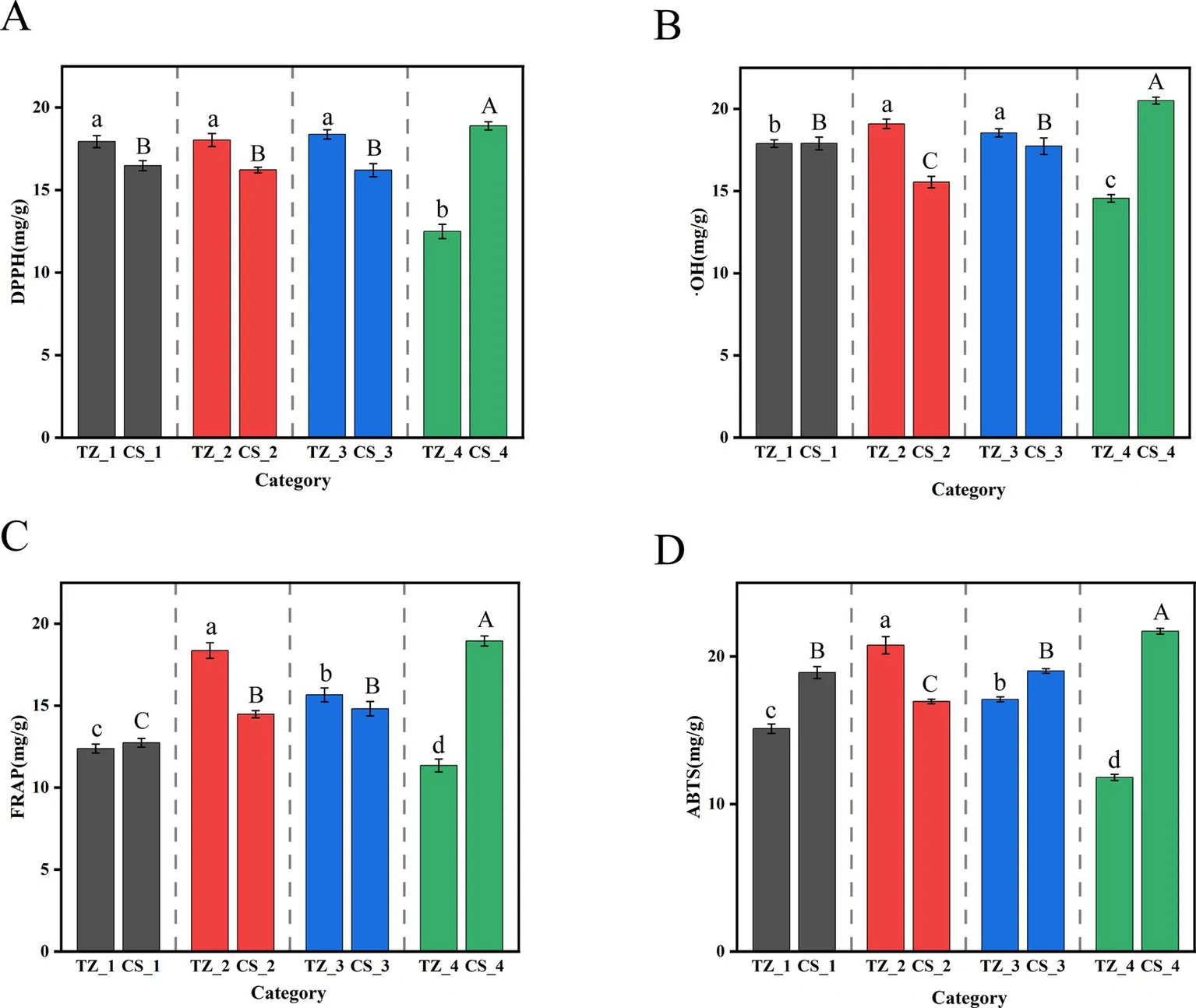
Antioxidant activity. **(A)** DPPH radical scavenging capacity. **(B)** Hydroxyl radical scavenging rate. **(C)** Ferric reducing antioxidant power (FRAP). **(D)** ABTS radical scavenging capacity. Data are presented as mean ± SD (*n* = 3). (a–d): different lowercase letters indicate significant differences among different harvest periods within the TZ group (*p* < 0.05, one-way ANOVA); A, B Uppercase letters indicate significant differences among different harvest periods within the CS group (*p* < 0.05, one-way ANOVA).

Figure 8 presents the antioxidant capacity of *P. lactiflora* roots under different planting systems and harvesting periods. Figure 8A illustrates the DPPH radical scavenging activity, with an order of CS-4 > TZ-3 > TZ-2 > TZ-1 > CS-1 > CS-2 > CS-3 > TZ-4. The peak activity of the TZ group occurred at the mid-harvest stage (TZ_3), while that of the CS group was observed at the final harvesting stage (CS-4).

Figure 8B depicts the ·OH radical scavenging activity, revealing an order of CS-4 > TZ-2 > TZ-3 > CS-1 > TZ-1 > CS-3 > CS-2 > TZ-4. The peak activity of the TZ group occurred at the mid-harvesting stage (TZ_2), and that of the CS group at the final harvesting stage (CS-4).

Figure 8C presents the FRAP reducing power of *P. lactiflora* samples, which ranked in the following order: CS-4 > TZ-2 > TZ-3 > CS-3 > CS-2 > CS-1 > TZ-1 > TZ-4. In the TZ group, antioxidant capacity initially increased and subsequently declined across the harvest stages, reaching its maximum at the mid-harvesting stage (TZ-2) and its minimum at the final harvesting stage (TZ-4). In contrast, the CS group exhibited a continuous increasing trend, with the highest and lowest FRAP values recorded at the final (CS-4) and early (CS-1) harvest stages, respectively.

Figure 8D presents the ABTS antioxidant capacity of *P. lactiflora* samples, with values ranked as: CS-4 > TZ-2 > CS-3 > CS-1 > TZ-3 > CS-2 > TZ-1 > TZ-4. The highest antioxidant capacities were observed at TZ-2 (TZ group) and CS-4 (CS group), whereas the lowest values were detected at TZ-4 and CS-2, respectively. The TZ group displayed an initial increase followed by a decline in ABTS, whereas the CS group showed a decreasing trend followed by an increase across the harvest stages.

The standard curves and *R*^2^values of all tests are shown in Supplementary Table 12. To evaluate the effects of planting pattern, harvest time, and their interaction on bioactive contents and antioxidant activities in *P. lactiflora*, two-way ANOVA was performed (Supplementary Table 13). To evaluate the effects of the planting system, harvest time, and their interaction on bioactive contents and antioxidant activities in *P. lactiflora*, two-way ANOVA was performed (Supplementary Figure 7). Harvest time showed significant main effects (*p* < 0.05) on all seven parameters, while planting system exhibited highly significant effects (*p* < 0.001) on paeoniflorin, total phenolics, total flavonoids, FRAP, and ABTS, but not on DPPH or ·OH scavenging activities (*p* > 0.05). The planting system × harvest time interaction was highly significant (*p* < 0.001) for all seven parameters, indicating that the intercropping–monoculture difference was strongly time-dependent, thus providing a statistical basis for stage-specific harvest strategies.

## 4 Discussion

Rhizosphere microorganisms exert a strong influence on the growth and development of medicinal plants. The rhizosphere forms the interface between the soil and the plant and serves as the key zone for the exchange of matter and energy flow within the ecosystem (Zhang et al., 2025). Root exudates and rhizosphere soil properties contribute to shaping these microbial communities, resulting in distinct community structures and functions (Luo et al., 2024). In addition to participating in biochemical soil processes, rhizosphere microbes affect plant growth and related ecological processes by producing phytohormones and signaling molecules (Kakar et al., 2018). The plants themselves can also select and adjust their rhizosphere communities through root exudates (e.g., organic acids and flavonoids), thus enriching functional groups such as salt-tolerant bacteria and enzyme-producing microbes. This recruitment helps to create a plant-friendly rhizosphere environment as it improves soil structure, helps nutrient cycling, and reduces salt stress (Wang et al., 2025; Cao et al., 2024; Zeng et al., 2022; Yuan et al., 2016).

This study employed 16S rRNA and ITS sequencing to investigate the differences in rhizosphere microbial diversity of *P. lactiflora* across planting systems and harvest times. Clear differences were observed in both alpha and beta diversity among the samples. Moreover, taxonomic make-up and functional profiles varied with the planting system and harvest stage. Our results agree with the literature on the impact of the growing system on rhizosphere soil microbes and potato yield (Wang et al., 2026). At the bacterial level, *Proteobacteria* was identified as the dominant phylum, while *Subgroup_2* was the most abundant genus after excluding unclassified taxa. For fungi, *Ascomycota* was the predominant phylum, and *Coniosporium* was the most abundant genus among classified taxa. These findings are largely consistent with previous reports on *P. lactiflora* (Wu et al., 2025). Moreover, the dominant fungal genera *Trichoderma* and *Talaromyces* are known to contribute to plant growth, health, and stress resistance (Zhu et al., 2023).

The buildup of secondary metabolites in medicinal plants varies with the ecological conditions, directly affecting plant quality (Yao et al., 2025). PCA results confirmed that the root metabolic profiles of *P. lactiflora* were clearly separated into clusters based on harvest period and cultivation mode. The number of differential metabolites between groups fluctuated across the harvest stages. These differential metabolites were primarily medicinal-related secondary compounds, including lipids, phenylpropanoids, and lignans. KEGG enrichment analysis indicated that the metabolic differences were mainly associated with metabolic pathways, particularly secondary metabolite biosynthesis, amino acid metabolism, and ABC transporter-mediated transport. These findings are consistent with a previous study on *P. lactiflora* metabolites (Wu et al., 2025), which integrated physiological, microbiome, and metabolomic analysis to distinguish among different varieties.

Endophytic microorganisms can alter metabolomic profiles by activating host biosynthetic pathways (Etalo et al., 2018). Network analysis revealed correlations between differentially abundant microbes and metabolites, suggesting that metabolites may shape microbial community assembly for environmental adaptation. We focused on flavonoid-associated taxa. Flavonoids were positively correlated with core microbes, implying potential interactions that require experimental validation. Phenolics and flavonoids from *Rosa chinensis* exhibit potent antioxidant activity (Cai et al., 2005), and variation in bioactive contents and scavenging capacities among onion varieties (Zhang J. et al., 2019; Zhang S. et al., 2019) aligns with our findings.

Differential metabolite dynamics mirrored the temporal trends of paeoniflorin, total phenolics, and total flavonoids. Phenylpropanoids serve as precursors of phenolics and flavonoids. Intercropping activated this pathway at early-to-mid harvest, enhancing bioactive accumulation, whereas monoculture allowed sustained accumulation to peak at late harvest. Late-stage intercropping suffered from shading and competition, suppressing secondary metabolism and reducing quality. Lipid divergence peaked at mid-stage, possibly modulating secondary metabolism.

Monoculture with late harvest (CS-4) maximized all quality traits and is recommended for quality-oriented production. Intercropping benefits were time-limited, with a sharp decline at TZ-4 likely due to shading and competition. CS-4 remains the priority, while intercropping at TZ-2/TZ-3 offers an earlier harvest with paeoniflorin levels superior to monoculture at the same stage. Mid-stage harvest with flexible adjustments according to annual climate is recommended for ecological intercropping.

PICRUSt2 and FUNGuild predictions are reference-based inferences rather than direct measures of *in situ* activity. In addition, soil physicochemical and microclimate variables were not measured in this study. Future research should integrate environmental monitoring to distinguish direct intercropping effects from indirect environmental modifications.

## 5 Conclusion

This study combined physiological measurements, microbiome profiling, and metabolomic analyses, with multivariate statistical methods to explore differences among *P. lactiflora* samples grown under different planting systems and harvest times. The results revealed that at the bacterial level, *Proteobacteria* was the dominant phylum, and among classified genera, *Subgroup_2* was the most common genus. For fungi, the dominant phylum was *Ascomycota*, while *Coniosporium* was the most abundant genus. Metabolomic analysis indicated that both the cultivation method and harvest time altered the metabolic makeup of the roots. This was deduced from the separate metabolic profile clusters, as well as the variation in the number of differential metabolites and types of unique metabolites among groups. Pathway enrichment analysis showed that the treatments primarily altered root metabolism by affecting metabolic pathways related to secondary metabolite biosynthesis, amino acid metabolism, and substance transport. We observed associations between microbial communities and metabolites, with flavonoids showing positive correlations with key microbial taxa, suggesting potential microbe–metabolite interactions. Monoculture with late harvest (CS-4) resulted in higher quality, while mid-stage harvest under intercropping (TZ-2/3) provided a balance between harvest and ecological benefits, with timing adjusted according to annual climatic conditions.

## Data Availability

All raw sequencing and metabolomics data generated in this study have been deposited in public repositories. The raw 16S rRNA and ITS amplicon sequencing reads have been submitted to the NCBI Sequence Read Archive (SRA) and are available at: https://www.ncbi.nlm.nih.gov/sra/PRJNA1499756. The raw untargeted metabolomics LC-MS/MS data have been deposited in the MetaboLights database and are available at: https://www.ebi.ac.uk/metabolights/editor/study/REQ20260723221958.
